# Telemedicine Experience for PrEP Care among PrEP-Eligible Women and Their Primary Care Providers during the First Year of the COVID-19 Pandemic in the United States

**DOI:** 10.3390/tropicalmed7100280

**Published:** 2022-10-02

**Authors:** Chen Zhang, Kevin Fiscella, Shelby Przybylek, Wonkyung Chang, Yu Liu

**Affiliations:** 1 School of Nursing, University of Rochester, Rochester, NY, USA; 2 School of Medicine and Dentistry, University of Rochester, Rochester, NY, USA

**Keywords:** telemedicine, PrEP and HIV care, providers and patients, COVID-19

## Abstract

(1) Background: During the two-year-long siege from the COVID-19 pandemic, a significant proportion of doctor visits transitioned from in-person to virtual. Scare evidence is available to assess the quality of patient-provider communication via the platform of telemedicine, especially for PrEP care within primary care settings. (2) Methods: Participants included 18 primary care providers and 29 PrEP-eligible women. Through content analysis and thematic analysis, facilitators and barriers embedded at different levels of telemedicine were identified and assessed. (3) Results: Women and providers reported pros and cons regarding their telemedicine experiences during the initial wave of COVID-19. Both groups of participants agreed that telemedicine visits were more convenient, efficient, and comfortable than in-person visits. However, without face-to-face interactions, some women felt less empathy, caring, and connected with their providers during virtual visits. Health providers expressed concerns with telemedicine, including patients’ privacy, lack of intimacy between patients and providers, and delayed lab work. (4) Conclusions: Our data indicate multi-level factors may affect telemedicine experience among PrEP-eligible women and health providers. Participants expressed concerns that may further entrench these long-existing health disparities in healthcare. Proactive efforts from policymakers, health professionals, researchers, and stakeholders are urgently required to tackle identified barriers and to pave the way for the new infrastructure that ensures health equity in society.

## 1. Introduction

Although a substantially large number of women continue to be at high risk of HIV transmission, these high-risk women report limited prevention options due to their vulnerable and disempowered status (e.g., discrimination, coerced unprotected sex, and mental health issues) [1,2,3]. As a key component of HIV prevention services, pre-exposure prophylaxis (PrEP; brand name Truvada^®^ (emtricitabine/tenofovir disoproxil fumarate) and Descovy^®^ (emtricitabine/tenofovir alafenamide)) is a useful prevention tool for individuals with high-risk of HIV infections. PrEP trials in women demonstrate that the gender-appropriate implementation of PrEP among high-risk women is both feasible and desirable [4,5,6]. PrEP is efficacious, safe, cost-effective, and particularly suited for women. Despite these potential benefits, PrEP uptake among women is very low [7]. The United States (U.S.) Center for Disease Control and Prevention (CDC) estimates that 225,000 HIV-negative women in the U.S. have indications for PrEP in 2018, but only 6.6% of them use PrEP [8,9]. Since the World Health Organization declared the novel coronavirus (COVID-19) outbreak as a global pandemic on March 11, 2022, officials of the U.S. have rolled out social distancing restrictions (ranging from “shelter in place” to “stay at home”) across most States of the U.S. [10,11]. The one-year-long “lockdown” order has paused many daily activities and impacted more than 310 million Americans [11]. Therefore, suboptimal PrEP use has been further compromised since COVID-19 started. Following the first month of the COVID-19 lockdown, an analysis of electronic healthcare data indicated that PrEP initiation decreased by 72.1%, and refill lapse increased by 278% as the result of low-perceived risks and inaccessibility of PrEP medication as the result of the lockdown order [12,13]. A recent study in the U.S. revealed that more than half of the PrEP-using women discontinued the PrEP regimen during the pandemic [14].

On the other hand, CDC’s surveillance data indicated that sexually transmitted diseases (STD) and HIV incidence spiked a few months after the pandemic started in the U.S., especially among under-resourced communities [15]. Furthermore, HIV incidence data were likely underestimated due to the limited medical resources and jeopardized testing services. The gap between increased HIV/STD incidence and decreased prevention services posed a challenge for healthcare professionals. Data collected across the U.S. showed a dramatic reduction in HIV and STD testing and prevention care during the pandemic compared with the pre-COVID period [16,17].

Telemedicine is an umbrella term that utilizes digital tools and information technologies to facilitate health care communications, diagnosis, treatment, and prevention on virtual platforms [18,19]. During the initial stage of the pandemic, telehealth applications were urged to be used in comparison to in-person healthcare visits. A recent study in the New York City of the U.S. indicated that telemedicine visits increased 683% one month after issuing the COVID-19 lockdown in urgent care settings [20]. Mckinsey’s report indicates that the overall utilization of telemedicine visits has plateaued at 38 times higher than that in the pre-COVID era in the U.S. [21].

At the time of the COVID-19 outbreak, healthcare in the U.S. had been separated into the “old” and “new” era of patient care [22]. Before the COVID-19 pandemic, the “old” era of healthcare practice consisted mostly of patients who met with their healthcare providers in person. During these visits, patients may encounter various scenarios that hinder their health-seeking behaviors. For instance, they may have to wait for weeks or months to get their first appointment, take time off from work for their appointments, or lack transportation to access healthcare [23]. These barriers were even worse among groups that have been economically and socially marginalized [24,25]. After the beginning of the COVID-19 pandemic, the “new” era of healthcare practice showed a dramatic increase in the utilization of telemedicine among patients and providers. Using telemedicine, healthcare can be accessed at any location (e.g., at home, at work) via multiple routes (e.g., telephone, computer, and mobile phones). However, for historically disadvantaged groups (e.g., individuals with low socioeconomic status, the elderly, and racial/ethnic minorities), the “new” practice of telemedicine may create another structural barrier to health equity or equitable care due to the limited access to technology or digital devices [22]. In addition, concerns regarding privacy, ethics, safety, empathy, and trust may pose unresolved and evolving challenges in the new digital era in the healthcare system [26]. The transition from traditional onsite visits to telemedicine virtual visits is beyond the simple “copy-paste” procedure [22,26].

To date, numerous research studies have thoroughly examined the role of telemedicine in healthcare, ranging from mental health and cancer treatment to lifestyle management [27,28,29]. However, few studies have examined the impact of telemedicine in HIV care including PrEP care during the COVID-19 pandemic. Furthermore, most available PrEP research focused on men who have sex with men [30], and few studies assessed PrEP care among women who disproportionately shoulder the HIV burden [31,32,33]. During the pandemic, women usually bear more family responsibilities (e.g., child care), which may further limit their health-seeking behaviors [31,32,33]. Therefore, in the current study, we employed a tailored conceptual framework to holistically assess the telemedicine experience in health care including PrEP care from key stakeholders (i.e., primary care providers and PrEP-eligible women).

## 2. Materials and Methods

### 2.1. Participants and Eligibility Criteria

Participants included healthcare providers and PrEP-eligible women. Eligible healthcare providers need to be 18 years or older, to primarily practice in New York State of the U.S., to practice as a primary care provider, to have prescription privileges, or to have prescription privileges under the supervision of another provider. PrEP-eligible women must be at least 18 years old, reside in New York State of the U.S., have had at least one visit with primary health providers in the past 12 months, and have PrEP indications based on the CDC guidance. All participants need to speak fluent English and be willing to provide consent.

### 2.2. Recruitment

Participants were recruited via multiple routes. Participants can either complete a brief screening over the phone or be sent by email a link to complete the screening via Research Electronic Data Capture (REDCap). For health providers, an Institutional Review Board (IRB)-approved recruitment message was distributed via the email lists for providers, online newsletters, and flyers. For PrEP-eligible women, information was distributed via the regional ResearchMatch^®^ portal to recruit volunteers who are willing to participate. Recruitment flyers were posted in clinics, Clinical Translational Science Institute listservers, and websites for participants.

### 2.3. Data Collection Procedures

The research team developed a semi-structured interview guide to explore critical barriers and facilitators regarding PrEP care in primary care settings and their experience using telemedicine during the first year since the COVID-19 outbreak. The interview guide was developed by exploring existing literature as well as by consulting with researchers, program evaluators, and key community members. Due to COVID-19 restrictions, all interviews were conducted via Zoom or phone. All interviews were recorded using the Health Insurance Portability and Accountable Act (HIPAA)-compliant digital recorders. Demographic measures were collected via the REDCap survey link at the time of the screening procedure. Interviews were semi-structured, with open-ended probes to elicit further information. Sample questions included “How does using telemedicine to provide PrEP care (or health care) differ from in-person PrEP care (or health care)?” (for health providers), and “Have you had any experiences with telemedicine and PrEP conversations during COVID?” (for women). All interviews were conducted by trained, experienced study interviewers using interview guides for eligible PrEP users and health providers. The average time of each interview was about 30 min.

### 2.4. Data Analytical Plan

Except for identifying information, all interview tapes were transcribed verbatim before analyses. All qualitative data were de-identified, with participant identification numbers used for analyses and sample tracking. Mixed methods, including content and thematic analyses, were used to analyze the transcribed narrative data. Following the five-step framework analysis, our approach included familiarization (describing potential themes), thematic framework (generating a nascent code/sub-code list), indexing (producing a final consensus code list), charting (critical thematic constructs to organize hierarchical themes), and mapping and interpretation (summarizing and interpreting) [34]. We employed an iterative approach to developing and refining a coding dictionary to enable data retrieval and analysis throughout the data collection procedure. Codes included theoretical constructs derived from the literature and the iterative qualitative data review. We coded transcripts using themes related to the central questions in our interview guides and new themes that emerged in the coding process. Content areas were then characterized by their emergent themes and some quantifications via the content-coding results. This process continued until no new themes emerged. Data were analyzed based on the theoretical framework. Two researchers independently read the code and categorized the qualitative transcripts to ensure inter-researcher reliability. Any discrepancies were resolved by discussion. Qualitative software (i.e., Atlas.ti) was used to facilitate the data analyses.

### 2.5. Ethics

Consent was an ongoing process that started when a subject was first informed about the study and ended when the subject’s study participation was completed. When potential participants contacted the research team by phone, the study staff explained the study and obtained their verbal consent to proceed with a phone screening using the phone screening script. A waiver of consent documentation has been requested for this step because it is conducted over the phone or by REDCap, keeping participants from signing this consent themselves. If screening was conducted via the REDCap survey link (by email), then an approved email was sent with the screening link.

## 3. Results

### 3.1. Participants

We included data that were collected from 18 primary care providers and 29 PrEP-eligible women in the current analysis. Specifically, 72.2% of the providers were female, and 94.5% reported “White” as their race/ethnicity. Among included providers, 55.6% reported: “family medicine” as their primary specialty, followed by “primary care” (38.9%), “internal medicine” (16.7%), “infectious diseases” (11.1%), and adolescent health (5.6%). Among the included PrEP-eligible women, their mean age was 38.1 years. The average number of sexual partners was 1.6, and 79.3% reported ever having condomless sex (Table 1).

### 3.2. Conceptual Framework

We employed a conceptual framework that was multifactorial in how each factor may affect telemedicine utilization in PrEP care [35,36]. At the technology level (level 1*)*, we explored how the design and characteristics of telemedicine hinder or facilitate PrEP care. At the stakeholder level (level 2), we examined providers’ and women’s attitudes, values, and experiences that affect their experience in telemedicine of PrEP care. At the context level (level 3), we scrutinized how primary care settings play a role in the telemedicine of PrEP care. At the policy level (level 4), we assessed how operationalization or reimbursement policies might affect the telemedicine of PrEP care in primary care settings. We reported critical findings from the current study using our framework as guidance to illustrate the complexity of the application of telemedicine in PrEP care in primary care settings (Table 2).

#### 3.2.1. Level 1: Technology-Level Factors Related to Telemedicine

1. Access to telemedicine devices and the internet

Stakeholders reported various capacities to access digital devices and the internet. Some providers were optimistic about the new application of telemedicine in healthcare as they believed that patients had some accessibility to telemedicine. One provider described, “*I think for a lot of patients, it’s really excellent and that it removes the transportation barrier. So some patients who no show a lot to clinic visits, you are able to reach them in their home and have nice conversations with them, um, and it’s, I think a big benefit.*” (PCP#40, physician, female, 30 years). On the other hand, providers acknowledged the limitations of telemedicine. For women without access or skills to use video, telemedicine over the phone may mitigate some of these barriers. Six women reported that they never had telemedicine experience. Some women used telemedicine for their healthcare. However, their devices were not equipped well for telemedicine. As one woman described:

“*my phone wasn’t really equipped to do that, so I don’t think on my end that it was not very good ‘cause my phone was very, um, glitchy. My device, I didn’t trust it. My phone wasn’t really equipped to handle that. It’s very horrible to hear, couldn’t really see her too well.*”

(woman #6, 36 years)

2. Digital Skills and digital literacy

Digital skills and digital literacy are the capacity to employ digital technologies to communicate and access information [37]. It is an essential contributor to health outcomes if it is used in healthcare [38]. In our study, patients reported different levels of digital skills to handle devices for telemedicine. Overall, younger patients were more likely to endorse the convenience of telemedicine for their healthcare. A 21-year woman said: “…*having my PCP be available, um, through telemedicine has also been very—real-really helpful. Um, I have found it, you know, pretty easy. I think, especially with everything being on technology and Zoom*.” (woman #35, 21 years). On the other hand, providers described older patients or patients who had fewer technology skills prefer the telephone over video as the platform for telemedicine. “*A lot of patient are really receptive to the telemedicine. They like it. Everything is, like, a virtual. We do have telephone counselors, but it’s usually more for patients who are a little bit older or not really good or tech savvy.*” (PCP#28, nurse practitioner, female, 36 years).

As providers observed, some patients had limited digital literacy and the capacity to adopt digital devices for their healthcare visits.

“*So some patients that can’t navigate Zoom are able to do that (Domixity: an application for telemedicine). I would say 50 percent can’t do it (Zoom). and the barriers there are either their ability, or they don’t—or they don’t have a phone with a camera, or they don’t have computers with a camera. just aren’t tech savvy.*”

(PCP#13, nurse practitioner, female, 65 years)

#### 3.2.2. Level 2: Stakeholder-Level Factors Related to Telemedicine

3. Acceptance level

Providers reported mixed feelings regarding the acceptance of telemedicine among their patients. Some providers reported positive feedback from their patients. The use of telemedicine eased the discomfort and anxiety caused by the pandemic. The acceptance level also varied among different providers. The majority of interviewed providers highly endorsed the convenience of telemedicine, while a few were reluctant to apply telemedicine in their practice. Providers acknowledged the pros and cons of telemedicine. Telemedicine reduced accessibility barriers, such as transportation and working hour conflicts. On the other hand, providers expressed some concerns, including “*you can’t see that physical thing”, “the fidgeting, the-the-the squirming… you might not see marks on their arms or lesions*”, and “*I can’t look in someone’s ears. I can’t feel their belly.”* That may cause delayed diagnosis and missed opportunities for treatment.

Most providers showed positive attitudes regarding telemedicine as they can sense patients’ reactions by reading their facial expressions, which were impossible if patients put masks on during in-person visits. “*one of the things I like about Zoom that I think is a benefit is they can see my face. So I don’t have a mask on. I don’t have a shield on. Um, so the—that even though it’s by camera, they can see that I’m smiling at them, or that I’m open to what they’re saying. I can see their face and read if they’re getting tired or if they’re uncomfortable*.” (PCP#1, nurse practitioner, female, 50 years)

A small proportion of providers showed high levels of frustration about the transition to using telemedicine; as one provider described, “I*-I-I was kind of frustrated because it seemed like there was almost a reluctance to, like, adopt telemedicine, um, because we have things like Zoom, we have things, um, like, built into the EMR that allow us to do video visits with patients. I was kind of, you know, frustrated that that wasn’t something that was part of my practice or that was part of, um, our practice in general… I did for the first couple months where I was at home, you know, feeling like a telemarketer calling people, um, for four hours straight. I don’t want that part of it*…” (PCP#15, physician, male, 28 years).

In addition, at least half of the interviewed providers showed evolving attitudes toward telemedicine: from refusal to acceptance. A provider described a dynamic flow of their patient volume since the pandemic started.

“*…we’re doing a big push now for PrEP virtual visits too, just to kinda get more and more people enrolled...Um, and it’s mostly for new patients, trying to get patients in outside communities. because of COVID, we definitely lost some PrEP patients. Just—or not lost, but they’re coming back now. And you know, they ran out of their prescription in April, and they just thought they couldn’t do a virtual visit... I think that we did lose some people for a few months, was just, like, lack of communication... they weren’t having sex, so they were, like, staying home. So they were like, oh, I’m not gonna be on PrEP. I think now I’m getting more people who I know who are recurrent patients, who are like, yeah, I stopped taking it, and I ran out in April, and I wanna restart.*”

(PCP#30, nurse practitioner, female, 36 years)

Among interviewed patients, their acceptance level of telemedicine varied based on their experience, ranging from extreme refusal to being comfortable with discussing PrEP. One patient was suspicious about the safety of the telemedicine and said “*that was my first one, and I told you I’m—you know, let that be the last because I don’t want to again. I didn’t feel comfortable or safe that my information was safe*.” (woman#6, 36 years). Another patient endorsed her recent telemedicine experience and reported feeling more supportive over the phone conversation: “*We’ve talked over the phone. I was fine with it… And I kinda felt like she was a little concerned. she addressed that … and listened—actually listened to me. So that felt like she was. when it was an in-person visit, you felt less supported than when it was a phone conversation; So you actually thought the phone conversation provided more positive experience*.” (woman #22, 55 years). A few patients shared their positive telemedicine experiences from different specialists, as a woman said “*visits with my G.I. doctor has been strictly over telemedicine, and some psychological services that I have seeked has also been either over Zoom or over the phone. It was almost more convenient ‘cause I didn’t have to drive anywhere. but for counseling services, it was fine over Zoom because it felt basically the same, if anything, it felt a little comfortable, um, ‘cause it wasn’t like their presence was, like, right in front of me. I’d be fine with it (PrEP care and discussion)*” (woman#32, 21 years)

Most patients reported mixed feelings about telemedicine; as one woman explained, “I think it (telemedicine) could have pros and cons. I think, um, telehealth could make it—could possibly make people more comfortable asking questions about it because it, it seems more private. So I think that could be a benefit. Um, I think one of the cons to, like, telemedicine would be it doesn’t feel as personal, so you don’t have as much eye contact with the, like, personal care provider. And so maybe you would trust them less or feel less care and less empathy. So it could have pros and cons. I preferred the face-to-face, definitely.” (woman #31, 20 years). About half of the women reported that telemedicine eliminated their transportation barriers and made healthcare very convenient, as one woman said: “when I did the mental health groups, those were on Zoom. think it’s practical. Um, and, uh, less time involved, especially during these times. Uh, you know, and so people like me that have to take the bus or, uh, maintain transportation, you know, uh, being able to still attend to my basic needs with my physician and being in my home is very, very convenient and helpful, and less fearful (of COVID).” (woman#7, 59 years).

4. Rapport and intimacy

The transition from in-person to telemedicine visits was easier among established patients, as there was already a rapport established from previous in-person communication. As a provider described, the application of telemedicine would be easier among established patients:

“*I mean maybe if you didn’t know the patient well, but the patients that I’m prescribing for I’ve known for years and years, so I don’t think it’s really all that challenging to read their body language or know, uh, get a sense for how they’re doing overall. If you’ve already established good rapport with that patient from years and years of being a doctor. I haven’t had too much difficulty with that*”

(PCP#23, physician, male, 46 years)

From patient’s perspectives, about 10 women mentioned that telemedicine made the healthcare experience less personal and intimate compared with in-person visits: “*I think one of the cons to, like, telemedicine would be it doesn’t feel as personal, so you don’t have as much eye contact with the, like, personal care provider. And so maybe you would trust them less or feel less care and less empathy… I preferred the face-to-face, definitely*.” (woman#31, 20 years). About seven women favored telemedicine as they felt more comfortable and supported than in-person visits.

5. Preference for artificial intelligence (A.I.) led Chatbot in healthcare

We further explored women’s preferences regarding the AI-led Chatbot in healthcare. Although half of the interviewed women had experiences interacting with Chatbot, some showed skeptical attitudes towards adopting Chatbot for their healthcare.

One patient shared her thoughts regarding whether a Chatbot can handle every question tailored to each individual as she said, “…*never a health bot. You know, I’ve used them for things like online purchases, but never for healthcare. I think if it was an option, and one of the other options was to talk to a person, I would be okay with it. I really don’t like, you know, in other situations of my life if I try to get ahold of a person and the only option is a bot, just because obviously they can’t answer every question. But it—as one option I would—I would be okay with it*.” (woman#11, 26 years).

Another woman expressed similar doubts on whether a Chatbot can handle nuanced situations “*I think I’d prefer to talk to someone, um, just because some of those questions can be a bit nuanced, and I don’t know—I wouldn’t know if the bot could actually see if—like, tell what I’m saying. Like, ‘cause it’s-it’s confu—medicine is confusing as it already is, so, like, I-I wouldn’t want it to misunderstand me, to lead me in the wrong direction. Um, I guess that’s fine because it’s just information. Like, that’s something that I can find online too*” (woman#19, 20 years).

Compared with patients with neutral attitudes towards the Chatbot, some women’s reactions differed significantly, ranging from high-acceptance level (e.g., “*I would love that. I probably would feel more comfortable with that than an actual person at times*” (woman#9, 33 years) to high-refusal level (e.g., “*I don’t like chat boxes [supposed to be “bot”]. I think that it’s a replacement for the human—the human contact, whether that’s audio or in person. speaking to a computer. Um, I-I-I tried to use those a couple of times, and the bot never—I’ve never had a successful encounter with a bot*.” (woman#7, 59 years). Younger women generally showed more positive attitudes and acceptance levels than older individuals.

#### 3.2.3. Level 3: Structural Factors Related to Telemedicine

6. Privacy and cybersecurity

About one-third of the participants expressed concerns regarding the privacy and confidentiality of the PrEP discussion during telemedicine visits. A provider explained, “*The only tricky thing I can see with doing a PrEP—having a PrEP conversation over the phone is you don’t know who else is in the room. Doing a telemedicine visit and speaking about someone’s sexual history and their risk for HIV is really a sensitive subject and not something that everyone wants to share with anyone else and it’s their right to do so. So I think it’s—that makes it harder*.” (PCP#2, nurse practitioner, female, 33 years).

On the other hand, some other providers indicated that patients were more comfortable discussing sensitive topics in their own space and time as one provider explained: “*You know, I think that there’s something to be said for when you’re asking some of these more sensitive questions, or the-the patient doesn’t necessarily have to look at you or be in the room, um, and then, you know, there’s privacy in going to a lab where, you know, maybe you don’t know the staff there, and, um, you know, I think that-I think that the person’s—patient comfort has increased with doing it kinda on their own time. It’s much easier to get somebody on the phone than it is to drag them into the office*.” (PCP#37, physician, female, 31 years).

Similarly, women reported controversial feelings about the privacy issue when using telemedicine. Some were concerned about the safety issues over the phone, while others considered telemedicine provided more private spaces for patients during healthcare sessions. As one of the women described, “*I think, um, telehealth could make it—could possibly make people more comfortable asking questions about it because it, it seems more private. Like, you’re—you can make the call from, like, your home where you’re in a safe place.*” (women#31, 20 years). Another young woman shared her experience regarding PrEP care using telemedicine “*for the time being, especially with COVID, like, telemedicine has been great. I think, for a lot of people, too, like, having your appointment in a space that’s really comfortable for you does make it a little more open for them. I think it’s probably a very positive component, um, especially talking about people’s sexual health. I think that can be a touchy subject for a lot of people, and I guess having that other l—that extra layer of security in their own home or comfortable space might make people even more open about talking about PrEP*.” (woman#35, 21 years).

However, a few women expressed concerns about the conversation being overheard by someone else. For instance, one middle-aged woman said: “*Um, I was just worried about where, um, she (the primary care provider) might be. You know, hearing the conversation, anybody.*” (woman#11, 52 years). Another woman shared the same concern: “*I think I would be, uh, fine talking to my O.B. on the phone as well or Zoom, whatever.was gonna offer just that. Like if it’s related to my sexual health I’d probably be way less inclined to have a Zoom where everybody—or a phone call.it wouldn’t be anymore convenient because I’d have to go find someplace—I can like go sit in my car or something. I think I’d be fine with most things unless it’s stuff I don’t wanna talk about out loud in a public setting*.” (woman#49, 26 years).

7. The immediacy of treatment and diagnosis

Providers and women reported some concerns regarding delayed lab work, treatment, and diagnosis. As one provider reported, there were missed opportunities for early diagnosis and treatment without immediate lab testing.

“*…in that particular case, um, you know, the patient—it was a phone visit, not a video. Um, and the patient described a rash that he had. And, um, based on the description I said, I’m pretty confident that you have syphilis. We need to get the labs done. The challenge is, is that if I had him in the office, I could’ve just treated him empirically at the time of the visit. PrEP via telehealth, which I’m sure is—I’m sure that’s going to be happening. I think it’s just, um, the missed opportunity to treat something at the time of a visit, or to catch something, um, early. You know, if-if I have somebody in the office and they have maybe some—a, you know, sore throat, you know, I can quickly get a swab to make sure that that’s not gonorrhea or chlamydia*.”

(PCP#14, nurse practitioner, female, 38 years)

Several women echoed the same concern about the delayed diagnosis, as one explained: “*I guess it just depends on if the doctor needs like a visual, I guess for the appointment. But if it doesn’t, then I guess just a phone call is fine. for example, like if someone has like a rash or something or a bump or something, and they need to like show the doctor I think that would be helpful to like show them, versus like describing on the phone.*” (woman #12, 22 years).

Furthermore, some providers discussed potential solutions for increasing accessibility and immediacy of lab work for PrEP care.

“*All of my, um, PrEP patients were done via telemedicine. So how that works is we do the same thing like we would do in the office. Of course, we can’t do the physical exam. We’re a little bit limited with that. And then we place orders so that we get bloodwork done. So we have places we they can go to, two locations. our clinic, what we were doin’ was, you know, we’re tryin’ to transition a lot of people with—to telemedicine for PrEP because it’s very flexible. And then have, like, every six months, have them do a in-person visit their complete physical.*”

(PCP#28, nurse practitioner, female, 36 years)

#### 3.2.4. Level 4: Policy-Level Factors Related to Telemedicine

8. Reimbursement for a telemedicine visit

Insurance companies may have different reimbursement schemes for different communication channels, which may affect providers’ preferences when providing care via telemedicine. In general, video-based visits (e.g., Zoom) can obtain higher reimbursement than phone-call only. One provider shared her experience: “*I prefer to do a Zoom visit because it will give me more information about a patient, um, so—and I think it’s a bit more personalized if you can see the person’s face and they can see my face. on the other end of the spectrum, is also billing reimbursement, that there’s currently much higher reimbursement for a Zoom visit than a telephone visit, um, so that the Zoom visit is definitely preferred*.” (PCP#39, nurse practitioner, female, 42 years).

## 4. Discussion

The “lockdown” procedure during the COVID-19 pandemic further compromises PrEP care in women who have been at risk of HIV infection as they are usually more vulnerable and bear more family responsibilities than their male peers [31,32,33]. With the popularity of telemedicine, we conducted one of the first studies to explore telemedicine application in PrEP care within the primary care setting among women with PrEP indications and their health providers. With the guidance of the conceptual framework, we reported key components embedded within various levels. At the technology level, women showed various accessibility and capacities of using telemedicine for their healthcare and PrEP care visits. At the stakeholder level, both women and providers reported different acceptance levels of telemedicine, ranging from refusal to comfort. At the structural level, stakeholders expressed concerns regarding privacy and confidentiality when using telemedicine. Furthermore, providers and women worried that the delayed lab work might miss the best diagnosis and treatment windows. At the policy level, insurance companies’ reimbursement schemes may affect providers’ preferences when using telemedicine in healthcare.

For PrEP care via telemedicine, some providers in the current study expressed concerns regarding the missed opportunities due to delayed lab work for diagnosing and treating patients with indications. On the other hand, a recent study conducted in a Boston community center indicated no difference in viral load suppression before and after the massive adoption of telemedicine among recipients of HIV care [39]. Therefore, a rapid and smooth transition from in-person visits to telemedicine may not significantly impact patients’ engagement in HIV care and their overall well-being. Some other providers considered telemedicine an excellent opportunity to discuss sensitive topics (e.g., sexual history, PrEP regime) with their patients, as individuals may feel more private and comfortable in the environment they were familiar with than in clinical settings. The reluctance to sexual health discussion has been reported as a long-existing barrier in healthcare [40]. Data from our study suggested that communication via telemedicine may provide a gateway for overcoming barriers impeding sexual health discussion between patients and providers.

Furthermore, participants reported varied accessibility and capacities of telemedicine for their PrEP care. Mainly, women reported limited access and understanding of telemedicine. Providers were concerned about the instability of telemedicine platforms and patient portals, low reimbursement rates, and lack of infrastructure and resources to absorb the new telemedicine infrastructure. Our findings echoed the identified “digital divide” [41], which was mainly attributed to access to digital devices and technology, digital literacy, and internet broadband coverage in existing studies [41,42]. The application of telemedicine cannot remove these long-built barriers without tremendous effort and time. Conversely, it adds an extra layer of complexity that arises from the rapid expansion of telemedicine to the existing structural barriers that contribute to inequitable care [43]. Despite these noticeable disparities, studies conducted in other countries (e.g., Brazil) and settings (e.g., rural areas) reported that the combination of self-testing kits, teleconsultation, and flexible lab locations could mitigate these barriers [44,45,46,47].

We endorsed several strengths in the current study. First, as one of the first studies to examine the application of telemedicine in PrEP care in primary care settings, we collected data from PrEP-eligible women and providers. With opinions from stakeholders from both sides, we depicted a comprehensive picture of PrEP care in the new era of telemedicine. Second, following the integrated conceptual framework, we have comprehensively assessed factors embedded at different levels to understand telemedicine in PrEP care implementation. Third, we employed the iterative approach to develop and refine a coding dictionary. This approach enabled us to familiarize data, develop a thematic framework, and index and interpret collected data to depict the holistic picture based on perspectives from key stakeholders [34].

On the other hand, we acknowledged a few limitations in the current study. First, due to the sampling scheme, participants were recruited from New York State in the U.S., which may guarantee limited generalizability of findings in the current study to other areas in the U.S. There is a significant disparity in PrEP care implementation between the Southern and Northern regions of the U.S. [48,49]. Further studies are strongly encouraged to explore regional-specific factors that may affect telemedicine application in PrEP care. Second, limited data were available to explore the barriers and facilitators of the application of Chatbot in PrEP care. As one of the promising compliments and alternatives in healthcare, we must carefully explore Chatbot-related topics in-depth in future studies [27,29,35]. Third, due to the scope of the current study, we did not assess how community-level socioeconomic characteristics (e.g., social vulnerability) impacted the applications of telemedicine in PrEP implementation in primary care settings among different groups [43]. In addition, women’s racial/ethnic data were not collected. Further studies will assess how racial/ethnic and hierarchical-level factors impact the transition of telemedicine across communities and groups. Fourth, although our sample size meets the requirement for sufficient “information power”, the relatively less-than-optimal sample size may not allow us to capture more nuanced and diverse input from the study participants, which warrants future studies to further explore this topic with a larger, more diverse sample. Lastly, these interviews were conducted in 2020 when COVID-19 first hit. We found many concerns about telemedicine from providers and patients as they had telemedicine as a new territory for them. As virtual service usage surged rapidly, many providers and participants became familiarized with online services after two and half years since COVID-19 first arrived. Thus, we recommend that future studies conduct interviews to see how their perceptions of telemedicine in PrEP care have changed. Moreover, particular populations are more likely to benefit from telehealth depending on their living location, socio-economic status, and physical health. Therefore, we suggest that future studies include providers and participants with diverse backgrounds.

## 5. Conclusions

In response to the COVID-19 pandemic, health care has rapidly adopted telemedicine including telePrEP care. Telemedicine has provided a convenient, efficient, and tailored modality in scenarios when in-person visits are not feasible. In the absence of research that examines factors that affect telemedicine experience among PrEP-eligible women and their primary care providers, our study contributes to the understanding of specific factors embedded at the technology, stakeholder, contextual, and policy level. Our findings could inform the future implementation of PrEP care among women with indications by increasing their accessibility and adherence to the PrEP regime.

## Figures and Tables

**Table 1:** Demographics and Key Characteristics of Included Participants.

Primary Care Providers (N = 18)		PrEP-Eligible Women (N = 29)	
Age (mean, * SD)	42.4(SD = 12.9)	Age (mean, * SD)	38.1 (SD = 15.1)
Year of Practice (mean, * SD)	9.0 (SD = 10.9)		
Gender (n, %)		Multiple partnerships (mean, * SD)	1.6 (SD = 1.1)
Male	4 (30.7%)		
Female	13 (72.2%)	Sex without condoms (n, %)	23 (79.3%)
Others	1 (5.6%)		
Race/Ethnicity (n, %)		Living with HIV-positive partners (n, %)	6 (20.7%)
White	17 (94.4%)		
Black	1 (5.6%)	Reported sexually transmitted infections in the past 6 months (n, %)	2 (6.9%)
Practice Places (n, %)			
Hospital-based	13 (72.2%)	Ever used substances (e.g., alcohol, cocaine, or other drugs) (n, %)	5 (17.2%)
Federally qualified health center	3 (16.7%)		
Group practice	3 (16.7%)	Ever injected drugs (n, %)	7 (24.1%)
Kaiser Permanente	2 (11.1%)		
Primary Specialty (n, %)		Ever had HIV testing (n, %)	20 (69.0%)
Family Medicine	10 (55.6%)		
Primary care	7 (38.9%)	Ever used PrEP (n, %)	3 (10.3%)
Internal Medicine	3 (16.7%)		
Infectious Disease	2 (11.1%)		
Adolescent Health	1 (5.6%)		

Notes: * SD: Standard Deviation.

**Table 2:** Summary of key findings from PrEP-eligible women and their primary health providers.

Key Domains	Sub-Category for Each Domain	Key Data from Women (n = 29)	Key Data from Providers (N = 18)	Major Similarities and Differences between PrEP-Eligible Women and Providers
Technology Level (e.g., capacity to access and use the telemedicine devices or technologies)	Access to telemedicine devices and the internet	Six women reported that they never had telemedicine experience	Providers reported various levels of patients’ access to telemedicine (from “a good proportion of patients lack of access” to “fully virtual visits at the clinic”)	Women and providers consistently reported various levels regarding the accessibility to telemedicine (e.g., from never tried, to using telemedicine all the time).
	Digital Skills and digital literacy	Six women did not report their feedback regarding digital skills	Five providers did not report revenant information	Among providers who provided data regarding patients’ digital skills, half of the providers reported their concerns regarding their patients’ technology skills to use telemedicine. Although 6 women did not provide information regarding their skills, the rest who responded indicated that they had the essential skills for telemedicine.
Stakeholder Level (e.g., women and providers’ attitudes toward telemedicine)	Acceptance level	Only eight women positively endorse telemedicine as an optimal health care option. The rest only used telemedicine as there were no other alternatives.	Only one provider explicitly expressed his frustration about telemedicine. All others showed positive attitudes toward telemedicine.	About two-thirds of the interviewed women expressed reluctance of using telemedicine as their healthcare option. Most providers showed enthusiasm for telemedicine as it may remove barriers such as transportation, access to care, and working hour conflicts.
	Rapport and intimacy	About 10 women indicated that they preferred in-person health visits. Seven women favored telemedicine as they felt more comfortable and supported. 12 women did not report relevant information.	Four providers explicitly considered that telemedicine provided more rapport for patients than in-person visits. Seven providers did not report relevant information.	Women reported a higher proportion of “lack of intimacy” than providers did. Providers considered that telemedicine worked better among long-term patients than new patients.
	Preference for artificial intelligence led Chatbot in healthcare	Six women explicitly expressed their reluctance of using Chatbot. Seven women expressed their willingness of trying Chatbot for PrEP or healthcare.	Providers did not report relevant information.	Some women expressed enthusiasm about Chatbot. However, no data for health providers’ opinions on Chatbots were available.
Contextual Level (e.g., how healthcare settings play a role in telemedicine)	Privacy and cybersecurity	23 women did not provide relevant responses. Three women expressed concerns about their privacy when using telemedicine. Another three women considered it safer via telemedicine than in person.	Nine providers did not provide relevant responses. Five providers expressed some concerns regarding the privacy and confidentiality of telemedicine. Two considered that patients would feel safer using telemedicine.	Both women and providers expressed similar concerns about privacy and confidentiality issues when using telemedicine.
	The immediacy of treatment and diagnosis	Only three women reported their concerns about the immediate lab work or capacity for diagnosis via telemedicine.	Three providers did not provide relevant information. Fourteen providers expressed concerns about the delayed diagnosis and postponed lab work. Five discussed how they got their patients’ lab work done under the framework of telemedicine.	Much more providers expressed their concerns about the delayed diagnosis and missed opportunities for treatments than women.
Policy Level (e.g., policies that may affect the application of telemedicine)	Reimbursement for telemedicine visits	No patient data are available.	Four providers expressed their concerns regarding the reimbursement procedure for telemedicine.	Providers indicated the insurance companies reimbursed much less for telephone visits than video visits, which may jeopardize the providers’ motivations to follow up with patients.

## Data Availability

The data presented in this study are available from the corresponding authors upon request. The data are not publicly available due to privacy concerns and the protection of sensitive information from the research subjects.
